# Focal Infection*Abstract from proceedings of the Michigan State Dental Society, April 13, 1920.

**Published:** 1920-09

**Authors:** Russell W. Bunting

**Affiliations:** Ann Arbor, Mich.


### FOCAL INFECTION *

BY RUSSELL W. BUNTING, D.D.SC., ANN ARBOR, MICH.

The past year has been for dentistry a year of introspection. The voices of her leaders as expressed in the publications of the year have not rung out with a clear, strong note of positive conviction, but rather have their expressions betrayed uncertainty and doubt. The great problems of oral infection and its relation to the health of the body have attained such prominence that they have overshadowed and dominated all other considerations in dental practice, and in the literature we see the strongest evidence that the dental profession is assiduously striving to learn what is the truth and to so order her procedures that they will be in conformity with the truth.

But the problem has proven to be so complex that so far its solution has baffled the keenest minds and no definitely positive course of action has been presented which can be adopted universally. In the writings upon this

* Abstract from proceedings of the Michigan State Dental Society, April 13, 1920.

subject, which have been voluminous (indeed, no issue of a journal is complete without one or more contributions), there is an almost unanimous agreement as to the grave significance of peridental infections to the general health, and among the various types of oral infection the greatest stress is laid upon the pulpless tooth and periapical invasions. The great prevalence of periapical disturbances about non-vital teeth has focussed the discussion upon the question as to whether a devitalized tooth may under any conditions be considered free from infection and safe to be retained in the mouth.

Among the medical and dental writers who have discussed this question, we see that there are three distinct classes: one, the empiracists, who say that no dead tooth is ever safe and stand unequivocally and unvaryingly for 100 per cent. vitality; second, the scoffers who doubt the significance of these periapical infections and their ability to disturb the general health; and, third, those who are conscientiously studying all phases of the subject, but who are attempting to keep to the middle of the road and arrive at a sane, conservative, rational policy that will successfully meet the situation. In certain sections of the county the empiracists have predominated and wherever their teachings have prevailed, the pendulum of opinion has swung so far away from the old-established methods of practice that thousands of teeth have been extracted and hundreds of patients have been made edentulous. As a conspicuous example of these the writings of Novitzky may be cited in which it is claimed by the writer that every devitalized tooth is infected six months after the root-canals have been treated and filled, and he denies the efficacy of any method of procedure to keep them sterile for more than that length of time. He, therefore, concludes that the retention of non-vital teeth is unsafe. These opinions have been widely quoted in the lay press as well as the professional journals.

On the other hand, there have appeared a number of statements by able members of both medical and dental professions in which it is claimed that sufficient evidence has not yet been advanced to warrant the wholesale extraction of all devitalized teeth and the attitude of empiracism, which has become so prevalent, is strongly condemned. Hartzell shows by statistics gathered from various sources that streptococcal diseases are a serious menace to human life and that the great majority gain entrance to the body through the oral tissues. But he states: ''The individuals who advocate complete destruction of dental organs, in order to rid the body of streptococcal growth, evidence profound ignorance. Wholesale extraction of the teeth will not rid the body of streptococcal growth. One might as well advise removal of the transverse colon to rid the body of colon bacillus. A much more sane method of cutting down the inroads of mouth infection is that of a vigorous systematic mouth sanitation together with the adoption of a diet planned to prevent intestinal putrefaction. Where teeth must be devitalized, such devitalization should be done under exactly the same kind of surgical asepsis as is demanded of surgery of the brain or abdominal cavity.'' He believes that the decision as to the removal of teeth should always depend upon an examination of the patient's vital resistance. ''A clinical picture, which would demand extraction, might be as follows: Early history of tooth and tonsil infection, swollen glands, draining the mouth and throat area, poverty of red cells, reduced hemoglobin, and a markedly increased or decreased leucocyte count.'' In cases in which the patient's resistance is high he would treat periapical and peridental infections, but when the resistance is found to be low he would extract the teeth, taking but a few at a time to reduce the anaphylactic shock.

E. C. Rosenow, in a contribution to elective localization, with special reference to oral sepsis, gives further proof of the validity of his claims as to the specificity of oral focal infections in their ability to produce systemic lesions. He states that in the clearing up of such infections the simple extraction of teeth is not sufficient, but rather that they should be removed surgically in order that all dead spaces in the bone might be eliminated at the same time. But he says, "If a person is perfectly well, has harbored for some years one or more devitalized teeth in which the X-ray findings are negative, there would seem to be no good reason for extraction. If, on the other hand, the person is suffering from arthritis, a heart or kidney affection, or some other form of disease for which other cause can not be found, such teeth should be removed. Owing to the reparative power of the cementum it would seem possible to devitalize teeth safely whose pulps are sterile and whose canals may be properly filled, providing that the operation is done in an aseptic manner. This should be done after the removal of other sources of infection and only in teeth of vital importance for restorative needs.

"In case of beginning infection of the pulp from decay, which has not extended into the periapical tissues, attempts at sterilization, and, if necessary, removal of the pulp and filling the canal, may be justified in some instances, but not until cultures have proved that the tissues are free from living bacteria, and only if the responsibility is shared conjointly by the patient and operator."

In a symposium of discussion directed toward the status of pulpless teeth which was held on the Pacific coast, W. C. Alvarez, a medical man, made a strong protest against the reckless extraction of teeth. He described the deplorable condition of mouths of patients whose teeth had been ordered out by physicians who, "quite oblivious to any possible value of the teeth to their owner, must have ordered their extraction simply because he believed it a panacea for most diseases. I believe that we have lost our heads over this thing and that the time has come to call a halt. Men have obtained such beautiful results in some cases by extracting teeth that some of them are now trying to explain most diseases on the basis of these focal infections. In practice they pull the teeth first and if the patient returns unbenefited they can then look to see what is the matter with him.'' He also speaks of those people who were ''cured'' by radical methods, but who after a period of relief are again going the rounds of doctors' offices.

He says further: ''Many of the dentists have become so frightend over the terrible results which they think must follow every root infection that they are refusing to fill any root-canals at all. They feel that the risk to life and health is so great that a man should immediately sacrifice every dead tooth in his head. Certainly the thousands of people who, for the last thirty or forty years, have been chewing contentedly on dead teeth (without signs of root infection), should be grateful that these radical ideas did not prevail when they were young.'' He concludes with the statement that, ''in view of the fact that most thorough removal of focal infections often fails to cure arthritis and other diseases, let us be more honest and conservative with our patients. Let us be careful what we promise them. Let us save serviceable teeth wherever possible. Above all, let us do unto our patients only what we would have done unto ourselves if their teeth were in our heads.''

Guy S. Millberry, in closing this symposium, says: ''Let us choose the middle of the road in this problem and assist nature first by endeavoring to prevent dental disease, and, second, by conserving and restoring to health those very valuable organs intrusted to our care, the human teeth.'' He also quotes A. D. Black as saying: ''I have a dead central incisor and a dead lateral incisor I have carried for thirty years and I hope to carry them as long as I live, for I have not been confined in bed for twenty-four consecutive hours in any one day since I was four years old.''

C. N. Johnson, from his broad clinical experience and observation extending back over fifty years, condemns the modern empirical attitude toward pulpless teeth. He says: "It would seem as if more than half a century of constant experience and observation on the part of the dental profession were to be cavalierly swept aside by a wave or two of the hand, and that the dicta of certain members of the medical profession followed by those of certain members of the dental profession were to override all the clinical experience obtained since pulpless teeth began to be filled. The impression has gone out from some quarters that the moment a tooth has lost its pulp it becomes a menace to the physical well-being of the individual, and should, therefore, not be tolerated in the mouth. This is so at variance with the clinical history of countless numbers of pulpless teeth that it can not be permitted to go unchallenged. Pulpless teeth have been of such incalculable service to people * * * that it will not do to sacrifice them on mere assertion. There has been altogether too much *ex parte* evidence on this subject, too much theorizing, too much panicky reasoning from illogical premises, too much reliance on laboratory experiments alone without studying sufficiently the things which actually happen in the mouth. This much is true, that if all the pulpless teeth of the past had been sacrificed, very many individuals who have enjoyed the blessings of adequate mastication would have been sadly crippled with all the consequent ills which follow a failure to properly perform this function."

E. C. Kells, in a paper entitled "The Crime of the Age," calls attention to the manifest errors in diagnosis and wholesale sacrifice of teeth based on radiographic evidence alone. He shows the limitations of the roentgenogram and condemns the diagnosis of peridental disease by the physician and roentgenologist. He also says, "That a dead tooth must necessarily be infected is the wildest of errors. That an infected root can not be rendered sterile is just as fallacious. Abscessed teeth, the roots of which were filled more than twenty-five years ago, that show apparently normal conditions in the skiagraph today, and the patients in perfect health, are clinical evidence of this fact which can not be controverted.''—*Journal National Dental Association.*

